# Acute Fulminant Myocarditis: A Bridge to Advanced Surgical Interventions With Veno-Arterial Extracorporeal Membrane Oxygenation

**DOI:** 10.7759/cureus.106572

**Published:** 2026-04-07

**Authors:** Imthiaz Manoly, Divya Ravikumar, Tagwa Omer, Fayaz Khazi, Obaid Aljassim

**Affiliations:** 1 Cardiothoracic Surgery, Dubai Hospital, Dubai Health, Dubai, ARE; 2 Cardiothoracic Surgery, Azerbaijan Medical University, Baku, AZE; 3 Anaesthesiology, Dubai Hospital, Dubai Health, Dubai, ARE

**Keywords:** acute fulminant myocarditis, cardiogenic shock, constrictive pericarditis, extracorporeal membrane oxygenation, inflammatory cardiomyopathy, pericardiectomy, severe biventricular dysfunction

## Abstract

Myocarditis is an inflammatory pathology of the heart muscle, often resulting from viral or bacterial infections and, very occasionally, due to drug toxicity. Acute fulminant myocarditis (AFM), the most severe subtype, is an underdiagnosed, life-threatening condition characterized by sudden worsening of cardiac function progressing to cardiogenic shock or multiorgan failure. It remains associated with substantial morbidity and mortality, often secondary to delayed diagnosis. Despite its profound presentation, AFM may demonstrate significant reversibility when recognized early and treated aggressively. Here, we present a challenging case of a middle-aged male patient who presented with AFM and required a multimodal approach in his successful management. The patient required veno-arterial extracorporeal membrane oxygenation (VA-ECMO) support as a bridge to advanced surgical procedures, including staged pericardiectomy and other supportive measures.

## Introduction

According to recent publications in global health databases, fulminant myocarditis accounts for about 10% of all myocarditis cases. Clinical presentation includes chest pain (usually mimicking acute coronary syndrome (ACS)), congestive heart failure (CHF), and fatal arrhythmias [1-3]. Management is mainly aimed at achieving hemodynamic stability and prevention of multiorgan failure [2]. Inflammatory pericardial involvement may complicate the disease course, occasionally progressing to constrictive physiology requiring surgical intervention [4-6]. In refractory cardiogenic shock, mechanical circulatory support such as veno-arterial extracorporeal membrane oxygenation (VA-ECMO) may be life-sustaining [7,8].

## Case presentation

A 45-year-old male presented to the emergency department with complaints of shortness of breath and substernal chest pain radiating to the left arm, associated with profuse diaphoresis. The patient reported feeling unwell for approximately one week, with fever and sore throat, prior to presentation at the Emergency Department of Dubai Hospital, for which he received a course of co-amoxiclav without full resolution of symptoms. According to the patient, symptoms began after a recent dental procedure.

He had a history of coronary artery disease and underwent staged percutaneous coronary intervention (PCI) to the left anterior descending (LAD) artery and right coronary artery (RCA) a year ago. His other medical history was significant for type 2 diabetes mellitus and dyslipidemia. He was an active smoker and had a high body mass index. He discontinued clopidogrel on his own six months prior to presentation.

**Figure 1:**
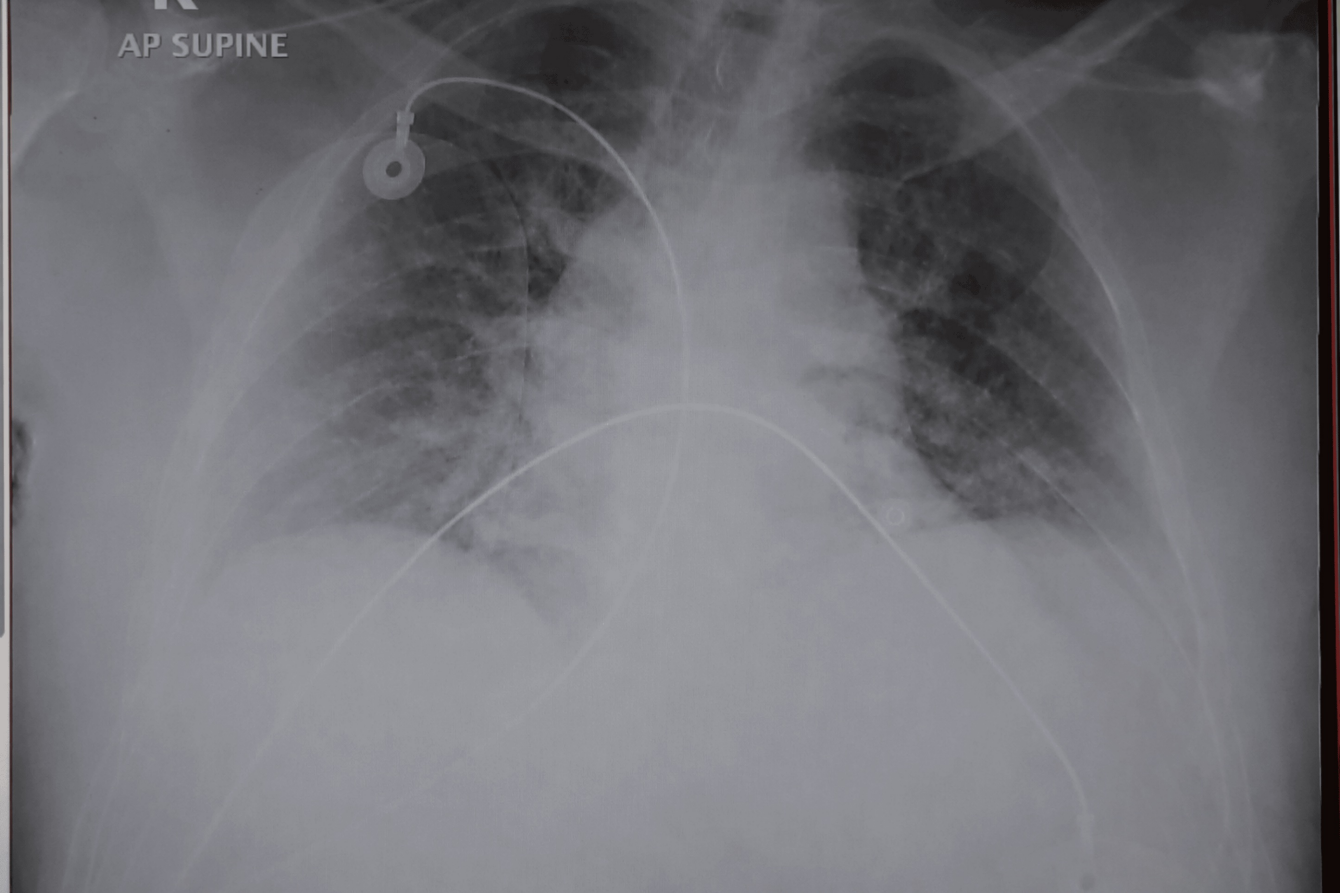
Chest X-ray of the patient on admission showing moderately bilateral congested lungs

**Figure 2:**
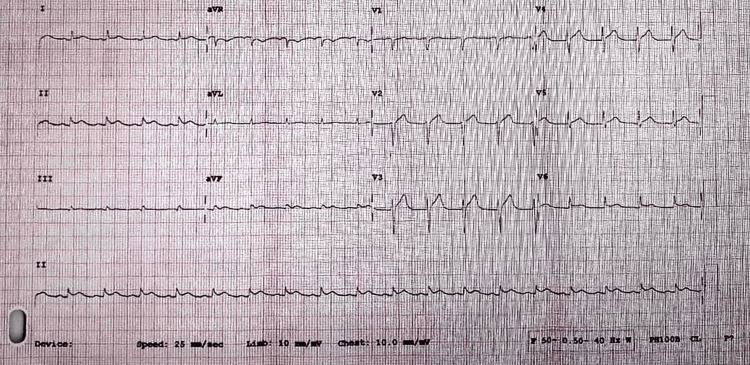
Electrocardiography (ECG) of the patient showing ST elevation on lateral lead and antero-inferior leads on admission

During the initial evaluation, the patient was noted to have a pericardial friction rub and diffuse ST-segment changes on electrocardiography (Figure 1), while a chest X-ray demonstrated bilateral congested lungs (Figure 2).

He was managed according to the ACS protocol in the coronary care unit and subsequently required endotracheal intubation for advanced respiratory support.

The patient remained hemodynamically unstable, prompting referral for advanced cardiovascular care, including consideration for extracorporeal membrane oxygenation (ECMO).

**Figure 3:**
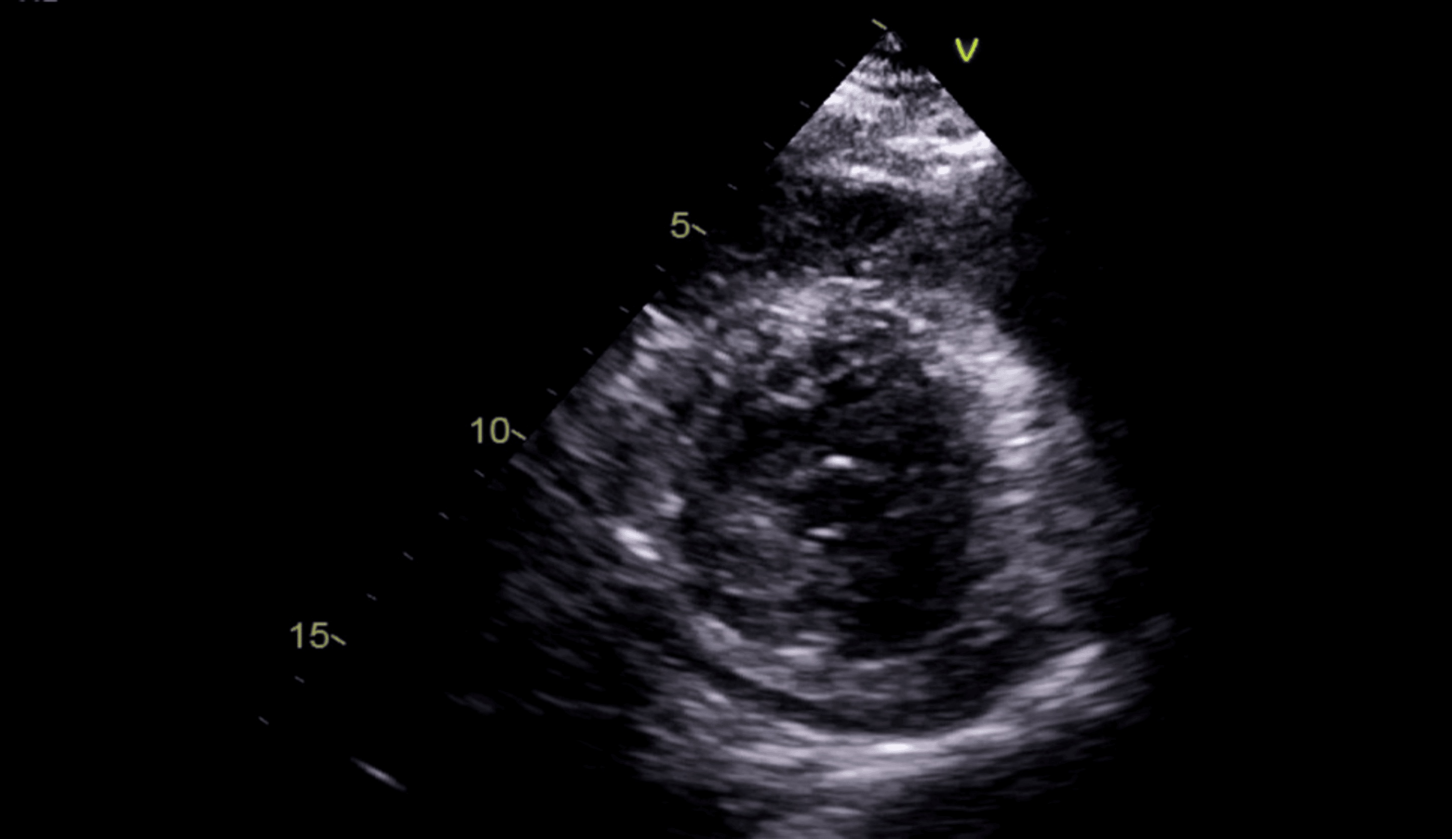
Echocardiography demonstrating pericardial effusion Initial transthoracic echocardiogram demonstrated global hypokinesia and pericardial effusion.

A transthoracic echocardiogram (TTE) performed on the subsequent day demonstrated severely impaired left ventricular systolic function, with an estimated ejection fraction of 15-20% (left ventricular hypokinesia), along with pericardial effusion (Figure 3).

The patient was tachycardic (heart rate 115/minute), with fused E and A waves, and evidence of elevated left ventricular end-diastolic pressure was noted. A mild-to-moderate pericardial effusion was noted without echocardiographic signs of cardiac tamponade (Figure 3). The inferior vena cava was normal in diameter with less than 50% respiratory collapse. He remained sedated, intubated, and mechanically ventilated.

A peripheral veno-arterial extracorporeal membrane oxygenation (VA-ECMO), utilizing a 21 Fr arterial cannula in the right femoral artery and a 23 Fr cannula in the left femoral vein, was initiated for cardiogenic shock. A distal perfusion cannula (8 Fr) in the right femoral artery distally was inserted to prevent lower limb ischemia, and an intra-aortic balloon pump (IABP) was inserted in the left femoral artery intraoperatively to reduce afterload and augment coronary perfusion.

Laboratory studies revealed moderately elevated ischemic biomarkers and markedly deranged inflammatory and septic markers (Tables 1, 2). He was started on tazobactam-piperacillin for a week. Over the ensuing days, there was a gradual decline in inflammatory and septic indices; however, the patient remained dependent on high-dose inotropic support to maintain hemodynamic stability and was unable to be weaned from mechanical ventilation or ECMO support.

**Table 1:** Summary of the results from the extensive blood tests. Note the rapid elevation of NT-proBNP and significant thrombocytopenia and deterioration of renal function. There was also a significant elevation of inflammatory markers over a short period of time. NT-proBNP: N-terminal pro–B-type natriuretic peptide; eGFR: estimated glomerular filtration rate.

Test	Reference range	April 3, 2024	April 6, 2024	April 10, 2024	April 14, 2024	April 26, 2024
Hemoglobin	13.0-17.0 g/dL	9.1	10	8.3	9.2	9.6
White blood cells	3.6-11.0 x 10^3^/mL	13.7	24.6	29.5	20.3	17.5
Platelets	150-410 x 10^3^/mL	130	102	80	25	45
NT-proBNP	<125 pg/mL	85.2	27,687	35,485	729.7	1,240
Procalcitonin	<0.05 ng/mL	146.40	36.60	10.20	1.29	0.36
Creatinine	0.70-1.20 mg/dL	0.72	1.57	1.64	1.49	1.12
eGFR	>60 mL/min/1.73 m^2^	115.5	55.4	52.6	59	83.1

**Table 2:** Summary of liver function panel SGPT: serum glutamate pyruvate transaminase; ALT: alanine aminotransferase.

Test	Reference range	May 2, 2024	May 5, 2024	May 9, 2024	May 23, 2024	August 29, 2024
Alkaline phosphatase	40-129 U/L	135	93	89	85	62
SGPT (ALT)	0-41 U/L	440	167	86	40	21
Total protein	6.6-8.7 g/dL	5.1	4.5	5.3	6.1	6.4
Albumin	4.4-5.1 g/dL	3.4	3.3	3.5	3.4	3.5
Globulin	2.8-3.4 g/dL	1.7	1.2	1.8	2.7	2.9
Bilirubin, total	0-1.2 mg/dL	2.83	2.67	2.81	1.10	0.30

**Figure 4:**
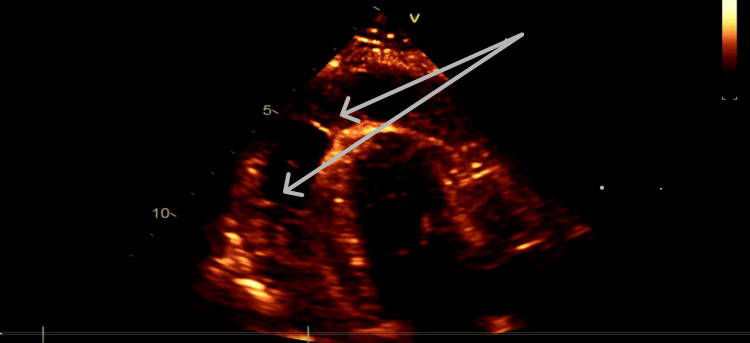
Echocardiography demonstrating pericardial effusion compressing the heart with fibrinous strands Global pericardial effusion with fibrinous strands compressing the myocardium

A repeat TTE performed five days later demonstrated left ventricular systolic function that remained severely impaired, with an estimated ejection fraction of approximately 25%. The visceral pericardium appeared thickened with an irregular surface along the right ventricular surface, raising concern for a possible organized clot. A large generalized pericardial effusion was present, measuring up to 2.6 cm at its maximal dimension (Figure 4).

**Figure 5:**
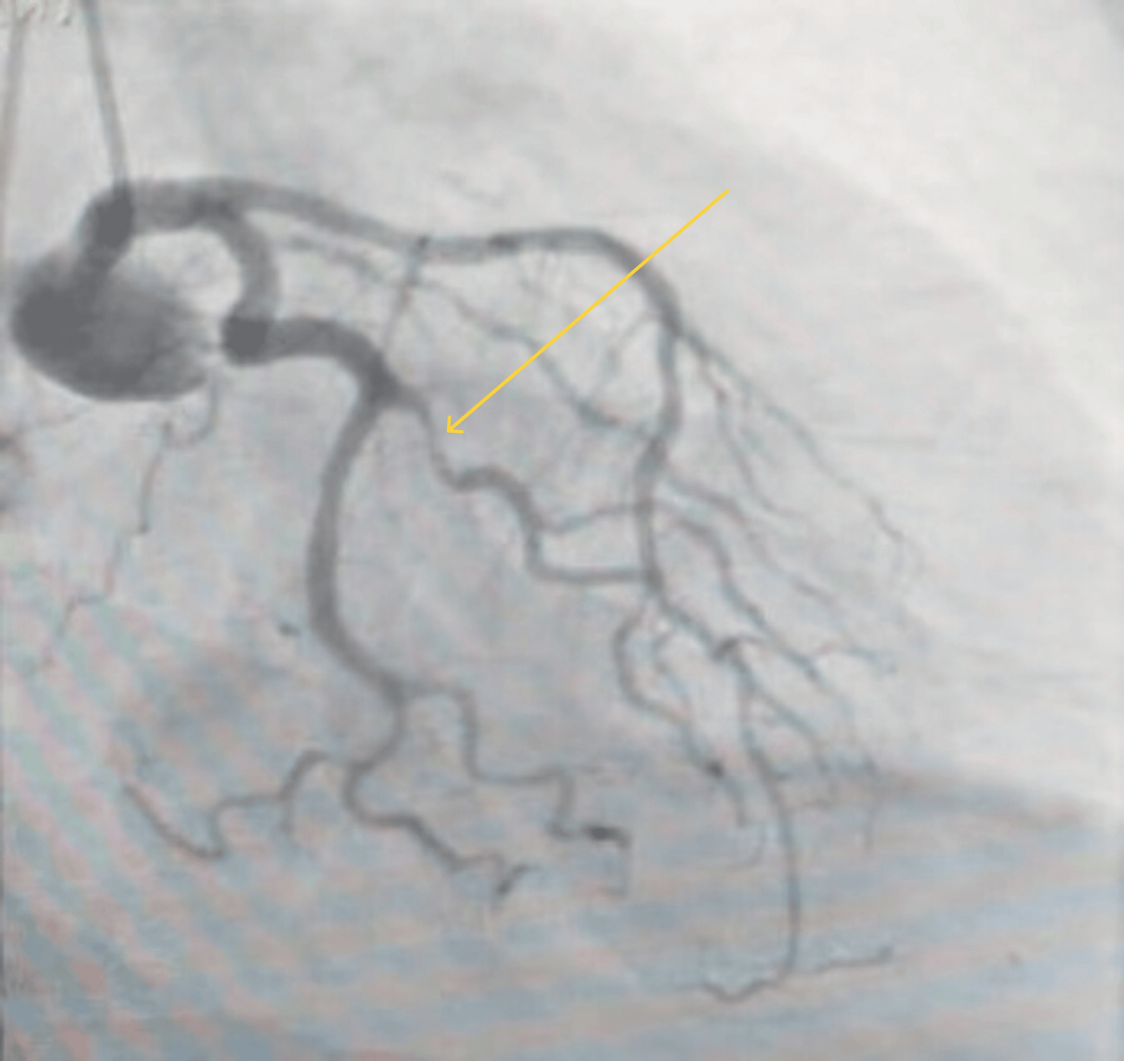
Coronary angiography demonstrating left coronary injection Coronary angiogram demonstrating stenosis in the second obtuse marginal artery.

**Figure 6:**
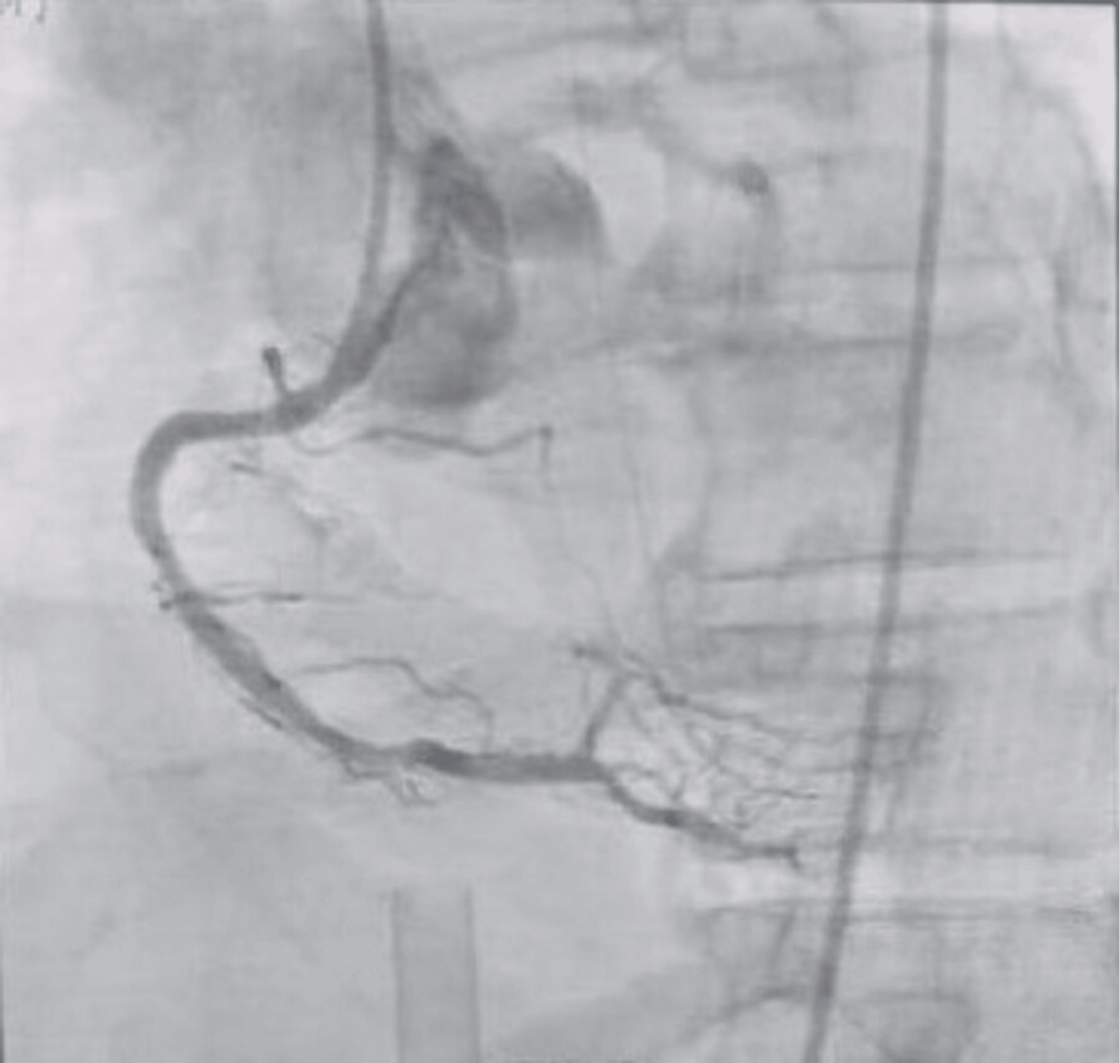
Coronary angiography demonstrating right coronary injection with patent stent

Coronary angiography (Figures 5, 6) performed on the following day depicted widely patent drug-eluting stents from the proximal to mid left anterior descending (LAD) artery, with Thrombolysis in Myocardial Infarction (TIMI) 3 flow and no evidence of offending pathology. The stents had been placed approximately 6-12 months prior. The left circumflex system showed an 80% concentric stenosis in the second obtuse marginal branch, with TIMI 3 flow. The right coronary artery (RCA) contained widely patent drug-eluting stents from the proximal to mid segments, also with TIMI 3 flow.

Attempted pericardial drainage in the catheterization laboratory was unsuccessful due to marked pericardial thickening and fibrinous effusion. The patient subsequently underwent a sub-xiphoid pericardial window. Intraoperatively, thick, brown, purulent-appearing pericardial fluid was evacuated. Microbiological analysis of the fluid was negative for organisms. Biochemical analysis revealed elevated triglyceride levels (100 mg/dL); however, the fluid was negative for chyle (reference range: <110 mg/dL non-chylous effusion; >110 mg/dL chylous effusion; <50 mg/dL pseudochylous effusion).

After one week of VA-ECMO support, there was no significant improvement in left ventricular systolic function. In addition, the right atrium and right ventricle demonstrated mechanical compression from a thickened pericardium and organized clot. Due to the lack of myocardial recovery and evidence of constrictive pathology, the patient was subjected to a right anterior mini-thoracotomy with partial pericardial resection, debridement, and suction of thick pericardial fluid. Although we initially proposed performing a sternotomy and pericardial resection, this was deferred due to severe thrombocytopenia.

Repeat transthoracic and transoesophageal echocardiography demonstrated biventricular systolic dysfunction with an estimated left ventricular ejection fraction of less than 15-20%. The patient continued with VA-ECMO, inotropic support, and IABP therapy.

**Figure 7:**
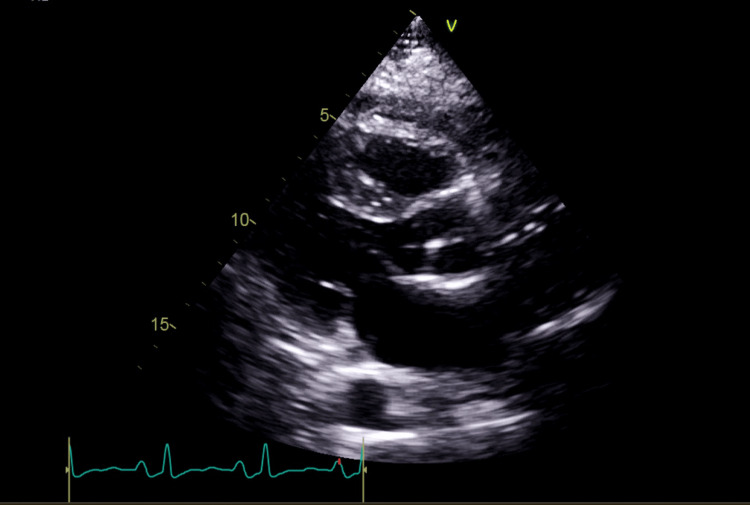
Echocardiography after pericardiectomy showing mild to moderate pericardial effusion without compressing the heart chambers

**Figure 8:**
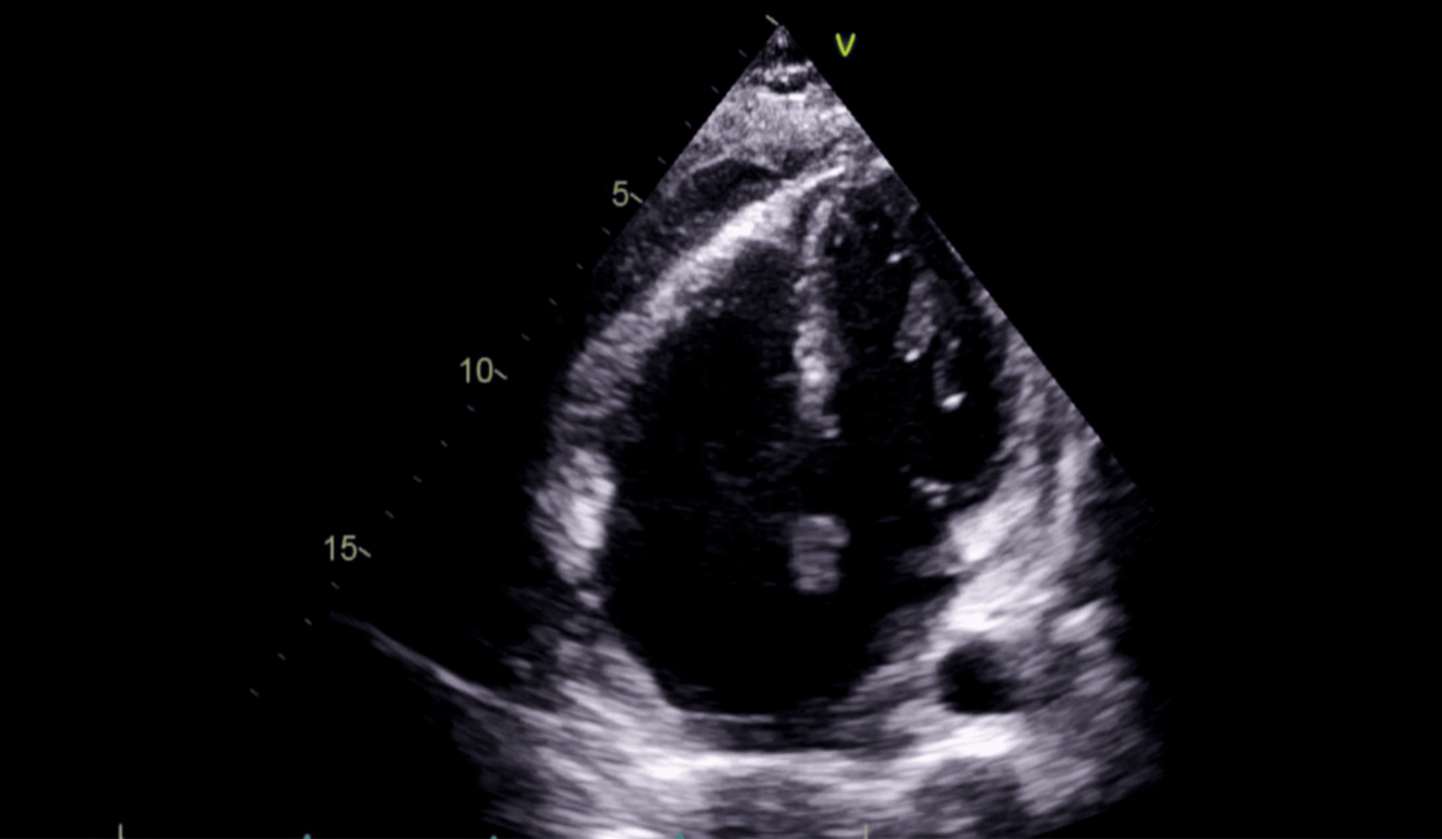
Echocardiography after pericardiectomy showing mild to moderate pericardial effusion without compressing the heart chambers

**Figure 9:**
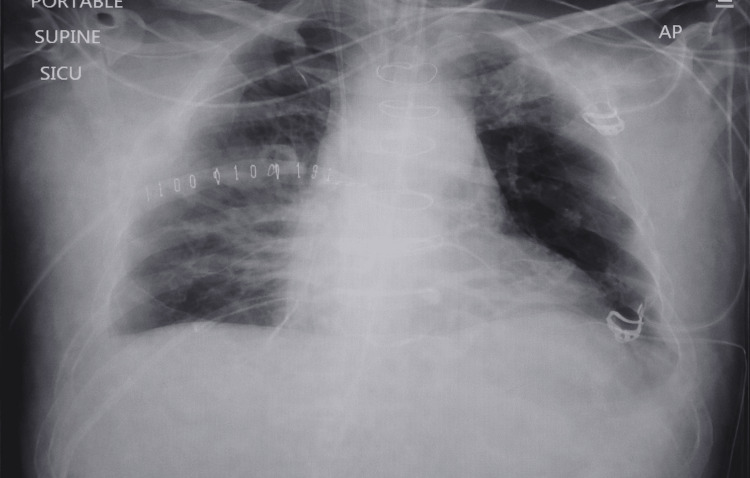
Chest X-ray of the patient post-operative demonstrating improvement of the lung infiltration

Heart transplantation was considered for this patient; however, it was not pursued due to significant deterioration of the clinical situation and pericarditis. Definitive management with a high-risk open pericardiectomy via midline sternotomy was hence performed following discussion with the family as a last resort. Postoperatively, the patient was returned to the surgical intensive care unit with ongoing VA-ECMO support, intra-aortic balloon counterpulsation, and mechanical ventilation (Figures 7-9).

**Figure 10:**
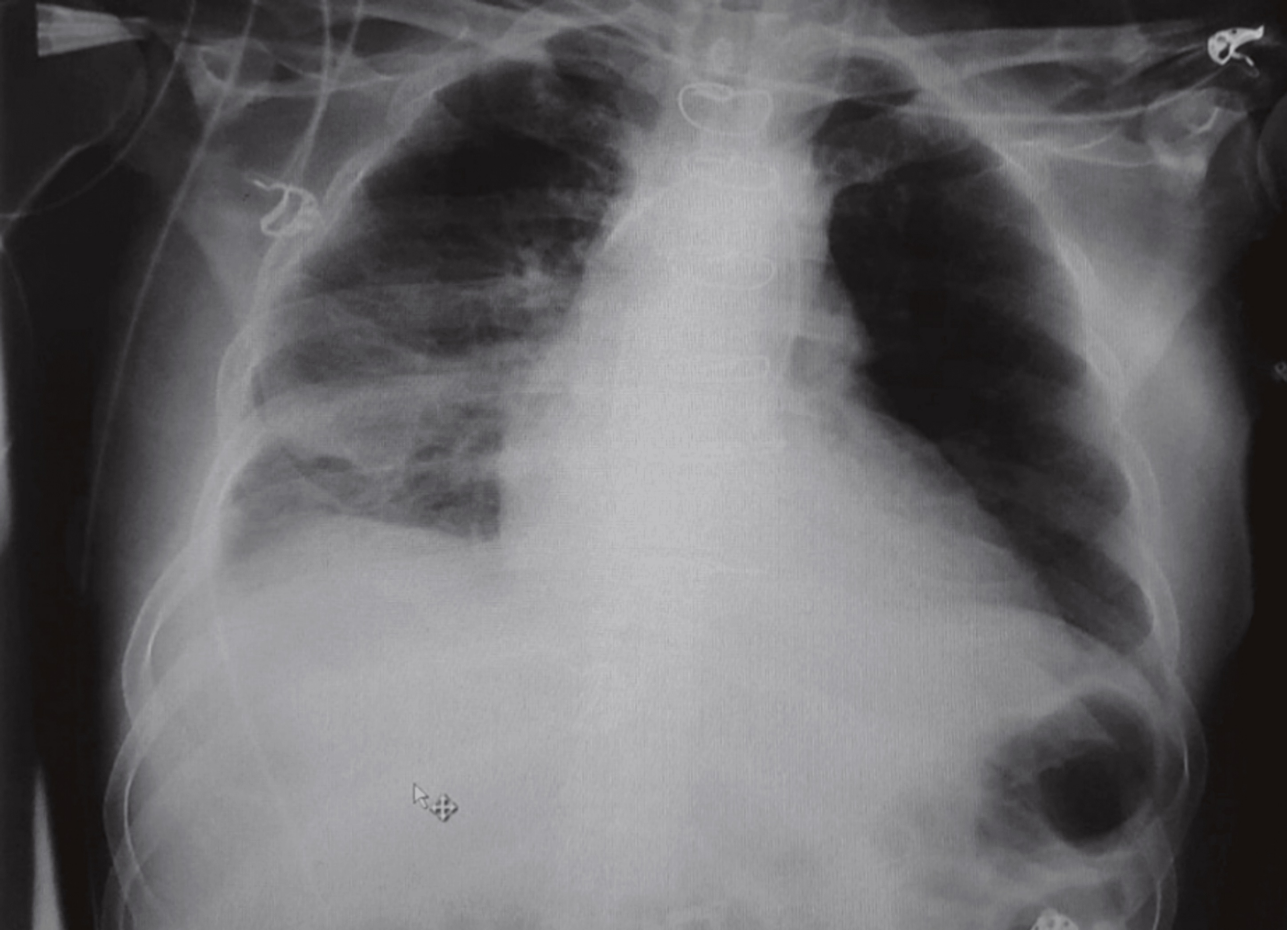
Chest X-ray of the patient on discharge showing lung infiltration on the right side; which improved compared to the previous ones

The patient showed progressive cardiac recovery, although the postoperative course was complicated by elevations in hepatic transaminases, renal indices, and septic markers, which were managed appropriately. The patient was subsequently decannulated from ECMO, and the IABP was removed after 13 days of its introduction (Figure 10).

Despite improvement in cardiac function, the patient developed acute kidney injury, which was unresponsive to medical management, necessitating initiation of dialysis the following day, which was continued for two weeks. Over the ensuing course, the patient gradually recovered from sepsis and shock liver with intensive supportive management.

Anticipating prolonged ventilatory requirements, a tracheostomy was performed a week later. As native urine output improved, the intensity of dialysis was reduced. With gradual improvement in respiratory mechanics, the patient was weaned from mechanical ventilation and transitioned to spontaneous ventilation mode.

The patient demonstrated improved global muscle power and remained on continuous (24/7) mechanical ventilatory support secondary to respiratory failure. The patient underwent both active and passive physiotherapy. Oral liquid intake was initiated and tolerated without reported complications. The patient was then symptomatically managed, including addressing some of the complications of prolonged immobilization, such as pressure sores.

Given the improving neuromuscular status, stabilization of respiratory support, and gradual enhancement of wound healing and functional capacity, the patient was referred to a long-term care/rehabilitation facility aimed at further improving muscular dynamics and providing continued supportive therapy.

## Discussion

Acute fulminant myocarditis (AFM) is a rare but rapidly deteriorating inflammatory disorder of the myocardium that is often misdiagnosed as ischemic heart disease due to overlapping clinical presentations [2,4]. The distinguishing feature is the abrupt development of severe global ventricular dysfunction leading to cardiogenic shock (Table 3).

**Table 3:** Differential diagnosis of Fulminant Myocarditis and Pericarditis HF: heart failure; MRI: magnetic resonance imaging; IVIG: intravenous immunoglobulin; ECMO: extracorporeal membrane oxygenation; LVAD: left ventricular assist device; VT: ventricular tachycardia; NSAIDS: non-steroidal anti-inflammatory drugs; SLE: systemic lupus erythematosus; RA: rheumatoid arthritis; ESRD: end-stage renal disease; MI: myocardial infarction.

Differential diagnosis	Associated conditions	Key clinical features	Key histologic/imaging findings	Treatment
Fulminant viral myocarditis	Viral infections (Coxsackie B, Parvovirus B19)	Sudden HF, hypotension, arrhythmias	Myocyte necrosis, lymphocytic infiltration (biopsy); MRI: edema	Inotropes, IVIG, steroids (select cases), ECMO/LVAD
Giant cell myocarditis	Autoimmune disorders, thymoma	VT, heart block, rapid progression	Giant cells, myocyte necrosis	Steroids + immunosuppressants; transplant
Necrotizing eosinophilic myocarditis	Drug reactions, hypereosinophilic syndrome	Flu-like symptoms, eosinophilia, cardiogenic shock	Eosinophilic infiltration + necrosis	High-dose steroids
Pericarditis (viral)	Viral infections, post-viral	Sharp chest pain, worse lying down, pericardial rub	Pericardial thickening/enhancement on MRI; no myocyte necrosis	NSAIDs, colchicine, +/- steroids
Autoimmune pericarditis	SLE, RA	Chest pain, pericardial effusion, systemic symptoms	Pericardial inflammation; serologic evidence of autoimmune disease	Treat underlying cause + NSAIDs/steroids
Uremic pericarditis	ESRD, dialysis patients	Chest pain, pericardial effusion	Fibrinous pericardium, inflammatory cells	Dialysis optimization, pericardiocentesis if tamponade
Post-MI pericarditis (Dressler's syndrome)	Autoimmune post-MI	Fever, pleuritic pain, pericardial rub	Pericardial inflammation weeks post-MI	NSAIDs, colchicine

Clinical presentation may vary widely from being asymptomatic to adverse events leading to cardiogenic shock and death [3]. The different types of myocarditis and pericarditis are diagnosed based on pathophysiology, timing of occurrence, and clinical course of the disorder (Table 3). Even though cardiac MRI is a very reliable non-invasive diagnostic tool, rapid deterioration of clinical condition and hemodynamic instability make it very difficult to use. Endomyocardial biopsy is the gold standard for the diagnosis of AFM.

In this patient, AFM was diagnosed in the context of underlying double-vessel coronary artery disease; however, there were a few salient features that distinguished myocarditis from ACS, namely the absence of regional wall motion abnormalities, global hypokinesia, and widespread ST changes on ECG. Due to his recent history of dental infection and significantly raised inflammatory markers at the time of presentation, bacterial myopericarditis was also considered as a differential diagnosis. In fact, he did develop pericarditis during the clinical course, as evidenced by a thickened pericardium.

This case study emphasizes the importance of early recognition and intervention in AFM complicated with pericarditis, particularly in patients with a history of ACS, as delayed diagnosis may result in rapid clinical deterioration. The use of ECMO provided critical hemodynamic support by maintaining organ perfusion and reducing myocardial oxygen demand during the period of severe ventricular dysfunction [5,6]. The decision to perform high-risk open pericardiectomy contributed to rapid decompression and enhanced diastolic filling, further aiding in circulatory stabilization [7,8]. These interventions proved pivotal in reversing the cardiogenic shock associated with fulminant myocarditis and pericarditis.

Even though the utilization of mechanical circulatory support devices, including ECMO and IABP, was done quite judiciously, a few associated complications were anticipated. These include lower limb ischemia due to VA-ECMO and IABP, coagulation consumption, and Harlequin syndrome (north-south syndrome). Close monitoring of the patient was therefore required to prevent the above-mentioned complications, in addition to titrating the anticoagulation regimen to balance between bleeding and thrombosis.

A pre-planned management strategy was not possible for this patient due to rapid clinical deterioration, and our approach was to mitigate the crisis by providing the most appropriate supportive measures to stabilize him. This included open pericardiectomy to decompress the heart after initiation of ECMO. Each new intervention aimed to either resolve an existing problem not fully addressed previously or manage a new complication. Effective communication with the family and multidisciplinary team involvement were key elements in decision-making in this critical situation.

This case demonstrated that aggressive multimodal therapy, including mechanical circulatory support and definitive surgical management, can reverse even profound myocardial dysfunction.

## Conclusions

AFM can be quite aggressive and can result in rapid deterioration of myocardial function, eventually leading to multiorgan failure. Even though the etiology is not conclusive, it often occurs after a viral infection. The clinical presentation could be imminent, and patients could go into malignant cardiac arrhythmias, cardiogenic shock, or even sudden death. Even though investigations demonstrate abnormalities, these may be non-specific, and the diagnosis was often confirmed on autopsy in earlier reports. Endomyocardial biopsy is the gold standard for diagnosing AFM. The ECG changes demonstrate low-voltage QRS complexes due to myocardial edema and, at times, show non-specific ST changes, which could raise suspicion of coronary occlusion. AFM could be differentiated from ACS with the help of echocardiogram, which demonstrates global hypokinesia as opposed to specific wall motion abnormalities. The management usually revolves around maintaining hemodynamic stability as soon as possible, with inotropes and possibly instituting mechanical circulatory support devices.

This case underscores the critical therapeutic value of ECMO as a bridge to either recovery or advanced surgical management, demonstrating timely aggressive mechanical circulatory support. The successful outcome in this case highlights the potential for full cardiac recovery, even in cases with severe initial presentations, provided appropriate therapeutic interventions are rapidly implemented, involving a multidisciplinary team.
